# Differences in the composition and predicted functions of the intestinal microbiome of obese and normal weight adult dogs

**DOI:** 10.7717/peerj.12695

**Published:** 2022-02-16

**Authors:** Pamela Thomson, Rodrigo Santibáñez, Camila Rodríguez-Salas, Carla Flores-Yañez, Daniel Garrido

**Affiliations:** 1 Escuela de Medicina Veterinaria, Facultad de Ciencias de la Vida, Universidad Nacional Andrés Bello., Santiago, Chile; 2 Chemical and Bioprocess Engineering, Pontificia Universidad Católica de Chile, Santiago, Chile; 3 Clínica Veterinaria Los Avellanos, Santiago, Chile

**Keywords:** Obesity, Gut microbiome, Canines, Microbiome function

## Abstract

Obesity is a multifactorial nutritional disorder highly prevalent in dogs, observed in developed and developing countries. It is estimated that over 40% of the canine population suffers from obesity, which manifests in an increased risk of chronic osteoarticular, metabolic, and cardiovascular diseases. The intestinal microbiome of obese animals shows increases in the abundance of certain members capable of extracting energy from complex polysaccharides. The objective of this study was to compare the composition and predicted function of the intestinal microbiome of Chilean obese and normal weight adult dogs. Twenty clinically healthy dogs were classified according to their body condition score (BCS) as obese (*n* = 10) or normal weight (*n* = 10). DNA was extracted from stool samples, followed by next-generation sequencing of the 16S rRNA V3–V4 region and bioinformatics analysis targeting microbiome composition and function. Significant differences were observed between these groups at the phylum level, with anincrease in Firmicutes and a decrease in Bacteroidetes in obese dogs. Microbiome compositions of these animals correlated with their BCS, and obese dogs showed enrichment in pathways related to transport, chemotaxis, and flagellar assembly. These results highlight the differences in the gut microbiome between normal weight and obese dogs and prompt further research to improve animal health by modulating the gut microbiome.

## Introduction

The gut microbiome represents an extensive catalog of microorganisms residing in the gut of animals (Marchesi & Ravel, 2015). Its composition is usually dominated by bacteria, with contributions of fungi, protozoa, and viruses (Vemuri et al., 2020). The gut microbiome has been directly or indirectly associated with host health in humans and other mammals such as dogs (Suchodolski, 2011; Alessandri et al., 2019). It has an impact on gut homeostasis, host metabolism, nutrient absorption, immune responses, and neurodevelopment, among others (Suchodolski et al., 2012; Heintz-Buschart & Wilmes, 2018; Alessandri et al., 2020; Siddiqui, Akbar & Khan, 2021).

The gut microbiome of healthy dogs is co-dominated by three phyla: Fusobacterium, Bacteroidetes, and Firmicutes (Middelbos et al., 2010; Hand et al., 2013; Chun Ju et al., 2020), with a lower proportion of Proteobacteria and Actinobacteria (Barko et al., 2017; Salas-Mani et al., 2018; Alessandri et al., 2020). In contrast to humans and other animal microbiomes, Fusobacteria is abundant in the gut of healthy dogs (Song et al., 2013; Vital et al., 2015; Bermingham et al., 2017).

Although the composition of the gut microbiota is stable during adult life, it is widely variable among humans (Guard et al., 2017). This microbial stability is also expected in dogs but only observed in the short term (Pilla & Suchodolski, 2020). Factors such as diet, drugs, and age are among the most important factors shaping the gut microbiome in dogs (Chandler et al., 2017; Gupta, Paul & Dutta, 2017; Kim et al., 2017; Li et al., 2017; Montoya-Alonso et al., 2017). A loss of microbiome homeostasis, or dysbiosis, has been linked to certain diseases such as inflammatory bowel diseases and metabolic disorders, among others (Pilla & Suchodolski, 2020). This alteration has been shown to be a consequence of the loss of key species or overgrowth of toxigenic microorganisms such as enterotoxigenic *Bacteroides fragilis* (Chandler et al., 2017; Gavazza et al., 2018; Craven & Washabau, 2019).

Obesity in dogs is a multifactorial disorder, with a prevalence greater than 40% in developed countries (Mao, Xia & Chen, 2013; Montoya-Alonso et al., 2017; Forster et al., 2018). Obesity is defined as the excessive accumulation of adipose tissue in the body, usually due to excessive food intake or the inadequate use of energy, causing a positive energy balance (Khera et al., 2019). Consequently, obese dogs suffer from a decrease in quality and life expectancy and an increased risk of developing diseases such as diabetes mellitus (DM), dyslipidemia, and cardiovascular disease, among others (Marshall et al., 2009; Clark & Hoenig, 2016; Chandler et al., 2017; Bjørnvada et al., 2019). At least in the last 50 years, the prevalence of DM has increased in dogs (Guptill, Glickman & Glickman, 2003; Heeley et al., 2020).

It has been observed that the relative abundance of Firmicutes and Bacteroidetes is altered in obese human subjects with an overrepresentation of Firmicutes, compared to lean subjects (Kasai et al., 2015; Haro et al., 2016; Coelho et al., 2018). Interestingly, in dogs this change in relative abundance can be observed in Firmicutes, Bacteroidetes, or Fusobacteria (Bermudez Sanchez et al., 2020). These taxonomic differences between normal weight and obese animals can contribute to the development and perpetuation of obesity (Li et al., 2017; Bermudez Sanchez et al., 2020). Proposed mechanisms include fat storage, regulation of energy metabolism, extraction of energy from short-chain fatty acids, increased low-grade inflammation, and impaired bile acid metabolism (Khan et al., 2016; Kieler et al., 2017; Xu et al., 2017; Garcia-Mazcorro et al., 2020).

Dogs, being domestic carnivores, take advantage of meat-based diets, and diet has a major influence on the composition of the gut microbiota (Wernimont et al., 2020). For instance, high fiber diets lead to an increase in the relative abundance of Firmicutes and a decrease in *Fusobacterium* and *Proteobacterium* (Bermudez Sanchez et al., 2020). A high-fat and low-carbohydrate diet enriches genera related to fat digestion, such as *Allobaculum* and *Parasutterella* (Kilburn et al., 2020). Partial weight loss can be achieved after dietary changes (Xu et al., 2017; Coelho et al., 2018; Apper et al., 2020).

The energy balance in animals is at a delicate equilibrium between energy consumption and expenditure (Stubbs & Tolkamp, 2006). The gut microbiota mediates changes in energy storage, in some cases leading to pathophysiological consequences in the short, medium, or long term (Ley et al., 2006). Few studies have addressed the impact of obesity in the canine gut microbiota, and the microbiome functions that could be altered in these animals are not well known. The goal of this study was to compare the composition of the intestinal microbiota in a group of obese and normal-weight dogs and predict what metabolic functions could be enriched or reduced in their microbiomes.

## Methods

### Subjects and inclusion criteria

This study was approved by the Bioethics Committee at the Veterinary Clinic Los Avellanos (Approval Certificate HCVLA-008). The study was performed at the same clinic, located in Independencia, Santiago, Metropolitan Region, Chile. Samples were collected during November 2020. Twenty dogs aged between 2 and 8 years old were sampled (Table S1). Animals were of any breed or sex and fed commercial diets (pellets) from different brands (Table S2). Inclusion criteria were for individuals who presented a normal clinical examination, physiological parameters (temperature, heart, and breathing rate), and no signs of gastrointestinal disease. Animals did not receive antibiotics or probiotics at least three months before the beginning of the study. All dogs had been spayed or neutered before the study.

All dogs were subjected to a complete clinic examination by a veterinarian. According to their body condition, ten normal weight dogs and ten obese dogs were enrolled. The body condition score (BCS) was determined based on a nine-point scale (German et al., 2009; Chun et al., 2019), based on palpation and visual inspection of the ribs, waist, bony prominences, the base of the tail, and abdomen. A one-unit increase in BCS corresponds to an approximate 10% increase in body weight (German et al., 2009; Chun et al., 2019). Animals with BCS values between 4–5 were considered normal weight, and dogs with BCS 8–9 were considered obese. Information regarding breed, age, and sex was obtained directly from each owner (Table 1).

**Table 1:** Animal data.

**Code**	**Age (years)**	**Breed**	**Sex**	**Weight (kg)**	**Body condition score**
1-N	5	crossbreed	F	31.8	5
2-N	5	crossbreed	F	31.7	5
3-N	3	crossbreed	M	13.3	5
4-N	4	labrador retriever	M	25	5
5-N	4	crossbreed	M	26	5
6-N	7	crossbreed	M	28	5
7-N	3	crossbreed	F	17.5	5
8-N	5	cocker spaniel	F	12	5
9-N	3	crossbreed	M	13.5	5
10-N	2	crossbreed	F	21.5	5
**Average**	**4.1 ± 1.4**			**22.75 ± 7.2**	**5 ± 0**
1-O	5	crossbreed	M	49	9
2-O	3	crossbreed	M	15	8
3-O	3	crossbreed	M	17.3	9
4-O	2	crossbreed	M	23	9
5-O	8	crossbreed	M	30.4	9
6-O	5	crossbreed	M	17	8
7-O	3	crossbreed	M	14.1	8
8-O	10	german shepherd	M	42	9
9-O	10	great dane	M	55	9
10-O	8	crossbreed	M	20.4	8
**Average**	**5.7 ± 3.1**			**33.5 ± 17.3**	**8.6 ± 0.5**

**Notes.**

F Female M Male

### Analysis of the gut microbiome

Stool samples were collected immediately after defecation and stored at −80 °C until processing. After thawed, 150 mg of each sample were used for total DNA extraction (Quick-DNA Fecal/Soil Microbe Miniprep Kit, Zymo Research, Irvine, CA, USA) using a Disruptor Genie device (Scientific Industries, USA). Fecal DNA samples were diluted to 20 ng/µl in nuclease-free water (NanoDrop 2000c; Thermo Fisher Scientific, Waltham, MA, USA ). DNA samples were submitted for Illumina MiSeq sequencing to the DNA Sequencing Services at Molecular Research (MR-DNA, USA). The variable region of the 16S rRNA V3–V4 gene was amplified using primers 341F and 785R (Klindworth et al., 2013), adding a barcode in the forward primer. The reaction was run for 30 cycles using the HotStarTaq Plus Master Mix Kit (Qiagen, Valencia, CA, USA). After amplification, the PCR products were verified on a 2% agarose gel. Several samples were pooled and purified using calibrated Ampure XP microspheres (Agencourt Bioscience Corporation, Beverly, MA, USA). The pooled and purified pooled PCR products were used to prepare a DNA library using the TruSeq DNA LT Sample Preparation Kit (Illumina, San Diego, CA, USA) following the manufacturer’s instructions. Sequencing was performed using the MiSeq platform (Illumina, USA).

### Bioinformatics analyses

The raw DNA sequences provided by the external service were analyzed employing the QIIME version 1.8.0 open-source bioinformatics tool (Caporaso et al., 2010). Each sequence sample was demultiplexed into individual files, and barcodes were removed from the 5′-end of each read (via demultiplex_fasta.py script). The processed sequences were uploaded to the European Nucleotide Archive under the project code PRJEB38793. Individual reads were assigned to bacterial taxonomy employing the DADA2 v1.10 R package (Callahan et al., 2016), following a modified procedure. Briefly, sequences were quality-filtered to remove undetermined base callings and trimmed down to 220 nucleotides before estimating the sequencing error model. The model was used to infer Amplicon Sequence Variants (ASV) (Callahan, McMurdie & Holmes, 2017) and those variants used to assign bacterial taxonomy with a Naïve Bayesian classifier (Wang et al., 2007) and the SILVA database version 132 (Quast et al., 2013; Yilmaz et al., 2014). The ASV abundance table was utilized to infer the abundance of metabolic functions and pathways with the PICRUSt2 python package (Douglas et al., 2020). Briefly, the PICRUSt2 software reconstructs a metabolism, first aligning an ASV to a reference tree that allows the selection of a reference genome and prediction of the gene content per ASV. Then, PICRUSt2 infers the abundance of metabolic functions and pathways employing the abundance of each ASV in a sample and the selected reference genome. Microbiome composition at the phylum level was assessed with the Shannon diversity index and the weighted UniFrac method (Lozupone et al., 2011) employing the scikit-bio python package (http://scikit-bio.org/). The weighted UniFrac was statistically assessed employing ANOSIM and PERMANOVA, using the scikit-bio software. Univariate analyses of the differences in the relative abundance of phyla, family, and genera were assessed with the non-parametric Mann–Whitney *U*-test (Mann & Whitney, 1947) and the DESeq2 R package (Lin & Peddada, 2020). Finally, multivariate analysis of the differences in the abundance of taxa, metabolic functions, and pathways was assessed with the Linear Discriminant Analysis (LDA) Effect Size (LEfSe) method (Segata et al., 2011). The LefSe method was performed employing the Galaxy server (Afgan et al., 2018) at https://huttenhower.sph.harvard.edu/galaxy/). In the case of the metabolic functions, the abundance of the KEGG orthologs (KO) and KEGG pathways were clustered and analyzed in a sample basis, and later, the contribution of each taxon at the genus levels and treatment was assessed only for the significant effect sizes of LDA (absolute value of the log10 LDA greater than 2) employing the Pearson Correlation Coefficient. Significance level for all statistical analysis was *p*-value <0.05.

## Results

This work analyzed the gut microbiome of ten obese (O) and ten normal (N) weight dogs, according to their BCS. The characteristics of the animals are presented in Table 1. Both groups were statistically similar in age and weight (Mann–Whitney *U*-test *p* ≈ 0.14 and *p* ≈ 0.09, respectively).

**Figure 1:**
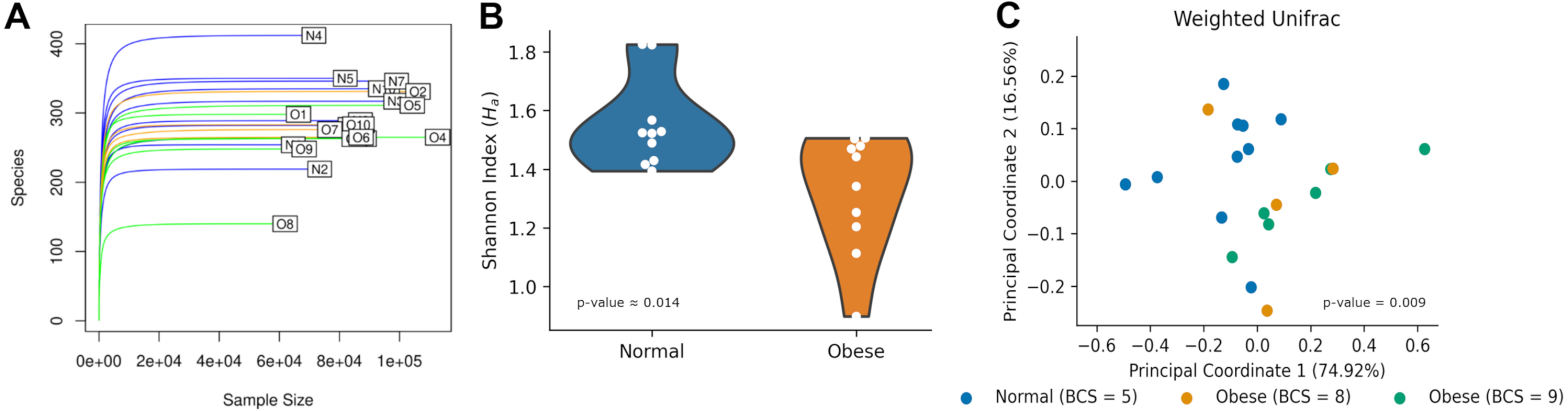
Microbiome diversity among normal and obese weight dogs. (A) Rarefaction curve. Number of identified Amplicon Sequence Variants (ASVs) as a function of the number of sequenced 16S amplicons. Blue lines identify samples of normal weight dogs, orange lines identify samples of obese dogs with a BCS of 8, and green lines identify samples of obese dogs with a BCS of 9. (B) Shannon index for each group. The violin plot shows all indexes and an estimation of the probability distribution of the data. (C) Principal Coordinate Analysis of the weighted UniFrac index for each sample. Each dot represents the UniFrac index, the proportion of relative abundance, and the similarity of phyla between the two samples. Indexes for normal weight dogs are shown in blue, while indexes for obese dogs are shown in orange   (BCS= 8) and green (BCS = 9).

After 16S rRNA sequencing of fecal samples, each sample contained between 100 and 400 ASVs (Fig. 1A). Rarefaction curves showed saturation indicating the sequencing depth was appropriate to describe the microbial composition. Alpha diversity using the Shannon Index, measuring the number of species and their abundances in each sample, was significantly different between both groups (N: 1.55 ± 0.15, O: 1.32 ± 0.20; Mann–Whitney *U*-test *p*-value ≈ 0.014; Fig. 1B).

Microbiome compositions in both groups were analyzed using the Weighed Unifrac beta diversity method. A PCoA plot of their compositions showed clustering of normal weight animals separated from obese dogs (Fig. 1C). The statistical assessment showed the beta diversity between obese and normal dogs was statistically different (ANOSIM R ≈0.179, *p*-value = 0.01; PERMANOVA pseudo-F ≈6.125, *p*-value = 0.009).

**Figure 2:**
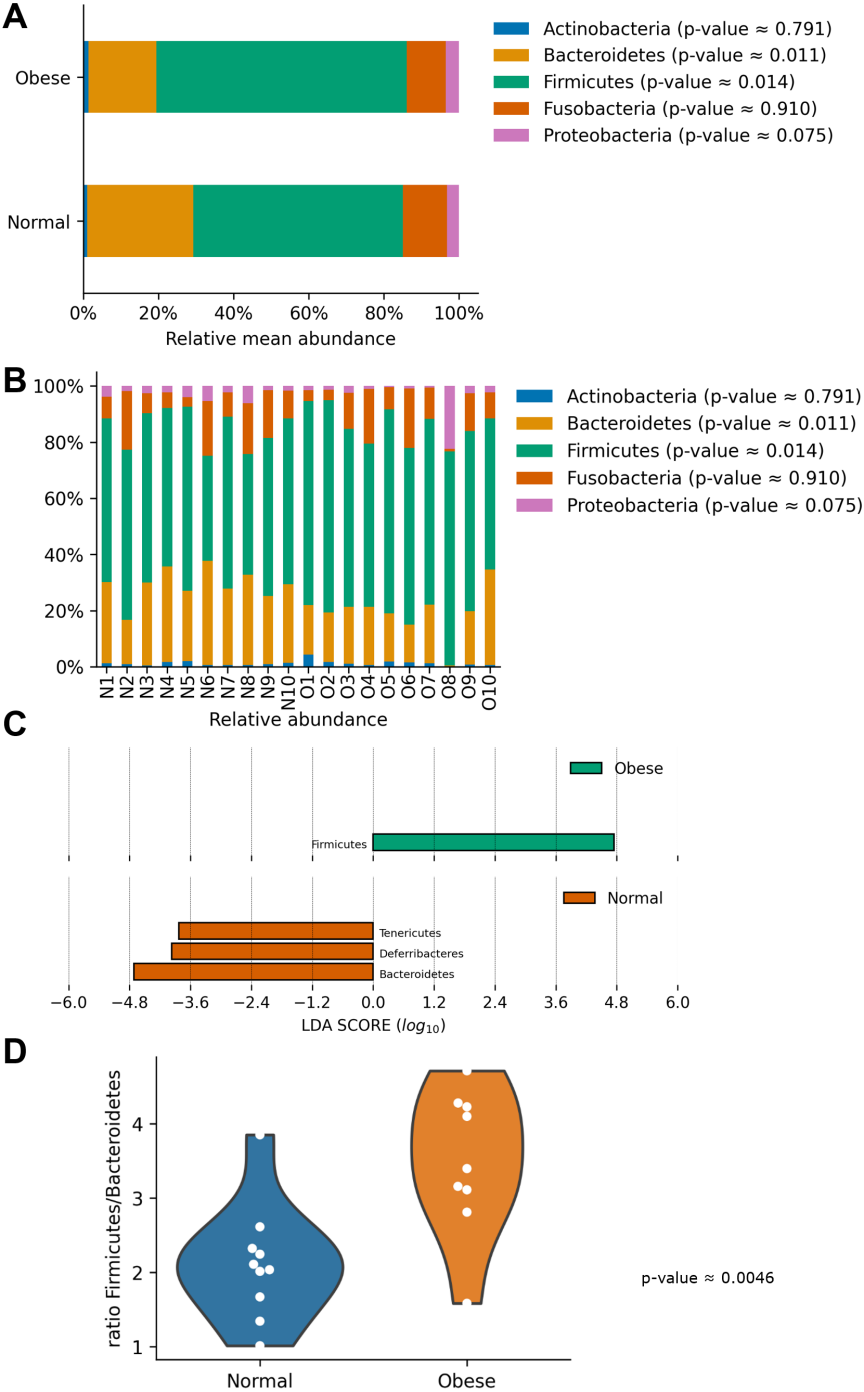
Relative abundance of representative taxa in dog gut microbiota at the phylum level. Relative abundance of representative taxa in dog gut microbiota at the phylum level. The figure shows the average proportion of Actinobacteria, Bacteroidetes, Firmicutes, Proteobacteria, and Fusobacteria (A) and each microbiome composition for each animal (B). *P*-values were obtained with the non-parametric *U*-test to determine differences. (C) Linear Discriminant Analysis Effect Size at the phylum level to identify major phyla enriched in obese and normal weight dogs. (D) Plot of the ratio Firmicutes/Bacteroides in both groups.

In both groups, the most abundant phyla were Firmicutes and Bacteroidetes, followed by Fusobacteria, Proteobacteria, and Actinobacteria (Fig. 2). Both groups presented significant differences in their microbiome composition at the phylum level. Compared to normal weight dogs, obese animals had a higher relative abundance of Firmicutes and lower abundance of Bacteroidetes (Mann–Whitney *U*-test *p*-value ≈ 0.014 and 0.011 respectively; Fig. 2A). Similarly, a LEfSe analysis at the phylum level showed significant enrichment of Firmicutes in obese dogs and significant enrichment of Bacteroidetes, Deferribacteres, and Tenericutes in normal weight dogs (Fig. 2C). Furthermore, the ratio Firmicutes to Bacteroidetes was significantly lower in normal weight dogs compared to obese dogs (0.28 ± 0.16 *vs.* 0.53 ± 0.21 respectively, *p*-value ≈ 0.004; Fig. 2D).

**Figure 3:**
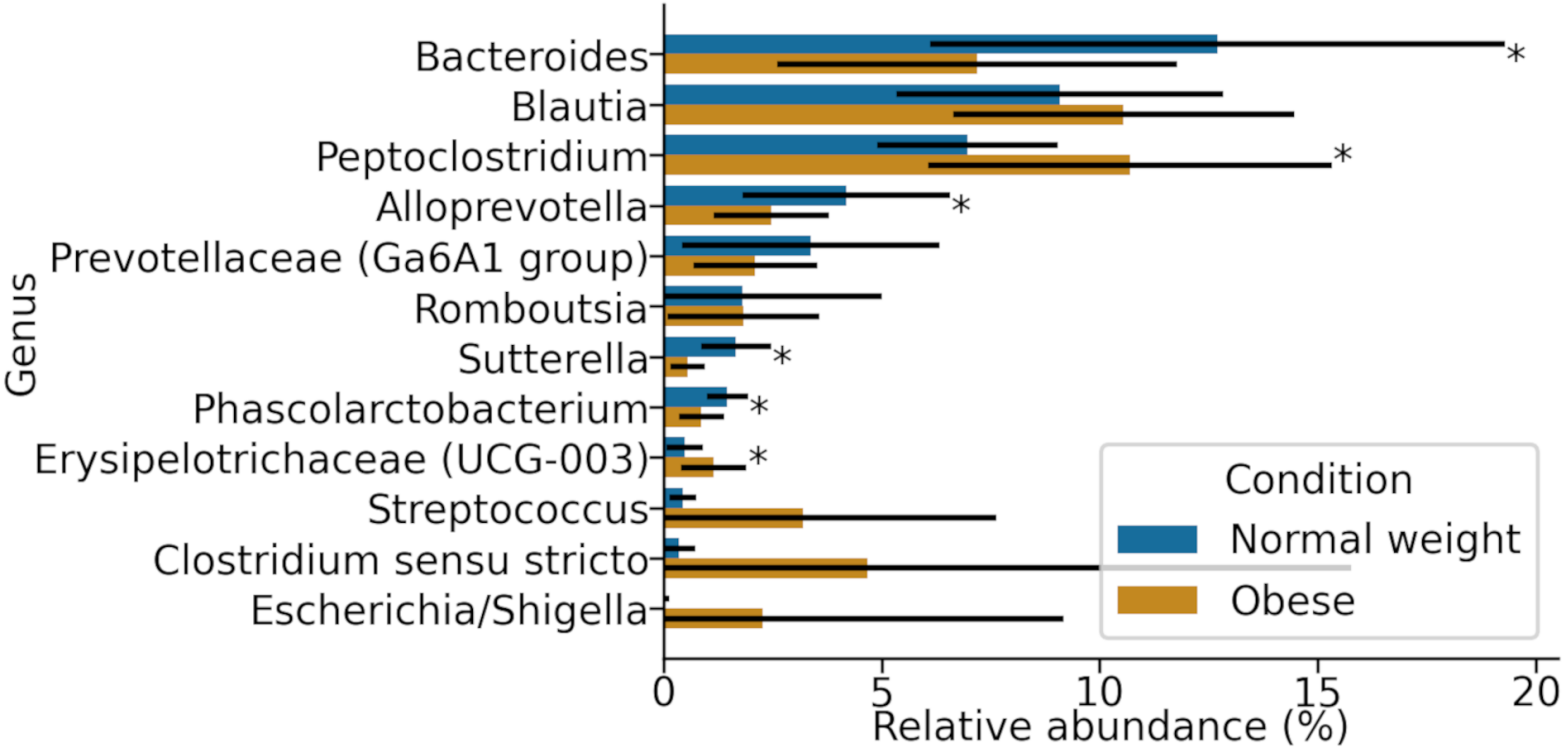
Most abundant genera in both obese and normal weight dogs. Data show the mean and standard deviation across all animals in both groups. Asterisks indicate *p*-value < 0.05.

At the genus level, samples in both groups were dominated by *Blautia*, *Bacteroides*, and *Peptoclostridium* (Fig. 3). Among these, significant differences in both groups were found in *Peptoclostridium* (DESeq2, adjusted *p*-value ≈ 0.048) and *Bacteroides* (DESeq2, *p*-value ≈ 0.048). In general, obese dogs had an increase in the relative abundance of *Peptoclostridium* and a decrease in *Bacteroides* genera (Fig. 3).

**Figure 4:**
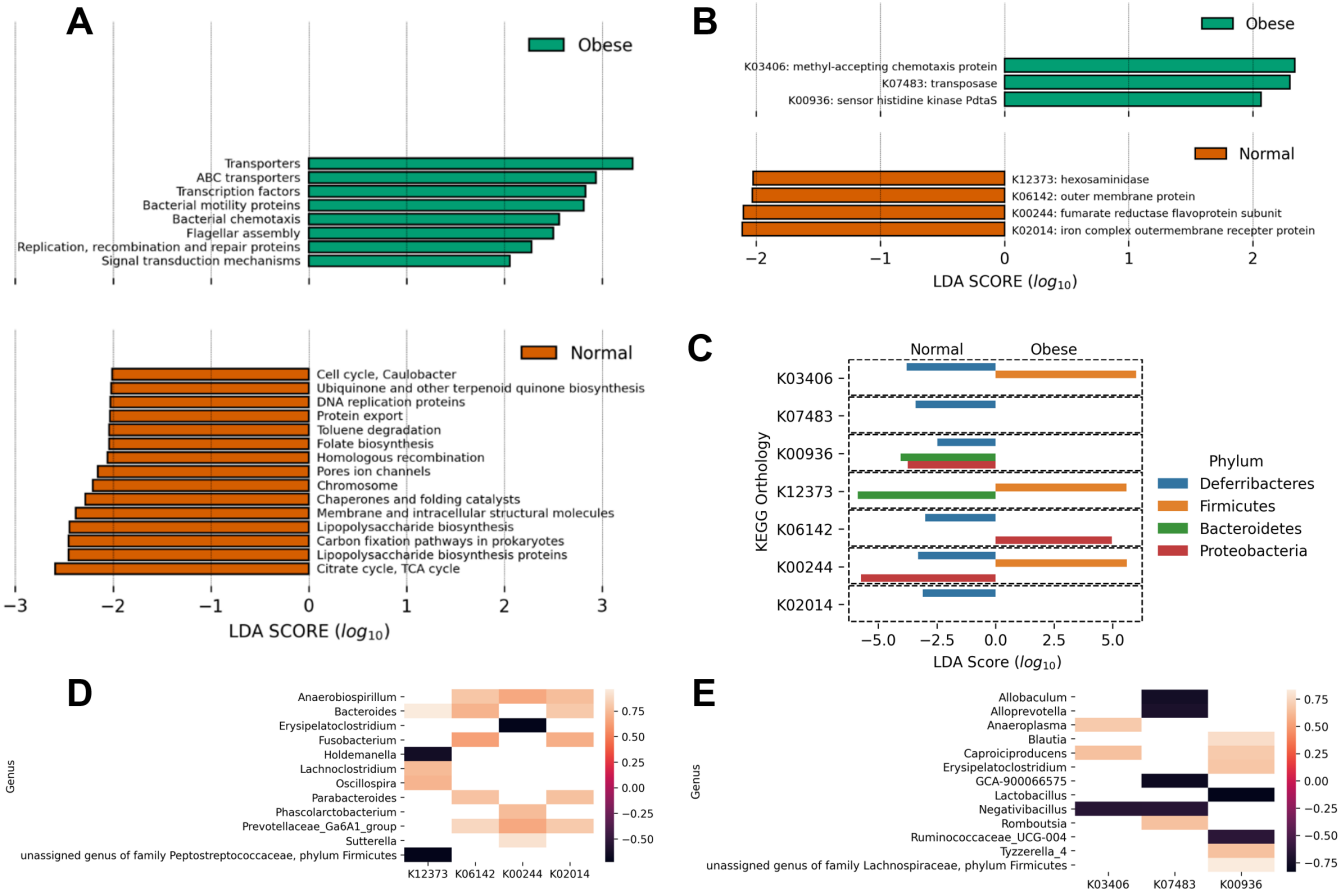
Predicted metabolic functions in the microbiome of obese and normal weight dogs. (A and B) Linear Discriminant Analysis Effect Size of the PICRUSt predicted metabolic functions abundance in obese animals. The top and bottom figures show the metabolic functions which abundance change most likely categorize the subjects in the study in normal weight (red) or obese (green). (A) KEGG pathways; (B) KEGG orthologs. (C) Determination of phyla contributing to the significant KEGG orthologs in (B) per-group basis. A LEfSe analysis per KEGG ortholog was applied to the abundances of metabolic functions per phylum, determining with phylum categorize the subjects in the study in normal weight or obese. (D and E) Pearson correlation analysis comparing the relative abundance of genera to the relative abundance of significant metabolic functions. (D) Significant correlations in normal weight dogs. (E) Significant correlations in obese dogs. KEGG orthologs are listed in (B).

Finally, using PICRUSt we predicted the abundance of major putative metabolic pathways in the gut microbiome of these animals and compared their total representation in both groups employing the LEfSe method (Fig. 4). A LEfSe analysis was first performed to determine genera enriched in both groups. We observed that *Peptoclostridium* was increased in obese animals, and several other genera were decreased (including Ruminococcaceae, Oscillibacter, and Parasutterella; Fig. S1).

Interestingly, obese animals showed an enrichment in KEGG pathways and orthologs related to motility (chemotaxis proteins K03406, flagellar assembly), as well as transport functions and two-component systems (Figs. 4A and 4B). On the contrary, normal weight animals showed a deployment in general biosynthetic pathways (terpenoids, folate, lipopolysaccharide, Fig. 4A), as well as hexosaminidases (Fig. 4B).

To detail the individual contribution of any microorganism to enriched or depleted metabolic pathways, the LEfSE analysis of the abundance of the KEGG orthologs at the phylum level was performed (Fig. 4C). The analysis revealed that the higher relative abundance of Firmicutes in obese dogs contributed to increases in chemotaxis proteins (*p*-value ≈ 0.005), hexosaminidase activity (*p*-value ≈ 0.016), and fumarate reductase activity (*p*-value ≈ 0.0005). These processes are related to respiration, motility, and degradation of host glycans. In addition, the analysis revealed that Bacteroidetes abundance in normal weight animals was responsible for the abundance of sensor histidine kinase function in obese dogs (*p*-value ≈ 0.003) and to hexosaminidase activity in normal weight dogs (*p*-value ≈ 0.016). This analysis was also performed at the genus level (Fig. 4D). *Anaerobiospirillum*, *Bacteroides,* and *Prevotellaceae* relative abundance correlated positively with 3 of 4 enriched KEGG orthologs in normal weight dogs (K06142, K00244, K02014; Fig. 4D). Similarly, *Caproiciproducens* relative abundance correlated positively with 2 of 3 identified KEGG orthologs by LEfSe in obese dogs (K03406, K00936; Fig. 4E). On the contrary, *Allobaculum*, *Alloprevotella*, *Lactobacillus*, *Negativibacillus,* and *Ruminococcaceae* relative abundance correlated negatively with the abundance of the three identified KEGG orthologs (Fig. 4E).

## Discussion

The gut microbiome has emerged as a factor shaping metabolic responses in animals, including canines (Bermudez Sanchez et al., 2021). In this study, we observed a significant decrease in Bacteroidetes and an increase in Firmicutes in obese dogs (Figs. 2 and 4). Bacteroidetes, together with Firmicutes, is one of the most abundant phyla in the canine intestinal microbiome, both in obese and normal weight dogs. A tendency of Bacteroidetes to decrease and Firmicutes to increase in obese dogs has been observed previously (Suchodolski, 2016). Interestingly, the ratio Firmicutes/Bacteroidetes has been shown to increase in dogs undergoing a high-fat diet accompanied by a reduction in insulin sensitivity and alterations in epithelial permeability (Moinard et al., 2020). This ratio has been shown to decrease in dogs under weight loss or inflammatory bowel disease (IBD) (Barko et al., 2017; Li et al., 2017; Bermudez Sanchez et al., 2020; Moinard et al., 2020). Notably, most of these studies have been reported in US and European countries, but only a few in other countries. In general, the evidence indicates a similar trend of increasing the Firmicutes to Bacteroidetes ratio in obese animals in different countries (Handl et al., 2013; Li et al., 2017; Montoya-Alonso et al., 2017; Bermudez Sanchez et al., 2020). However, further studies and proper statistical comparisons are required to determine the effect of geography on the gut microbiota and obesity in dogs. The Firmicutes/Bacteroidetes ratio imbalance has also been observed in obese humans, being reversible after dietary interventions (Ley, Turnbaugh & Klein, 2006). While obesity is a multicomponent disease and dogs were classified as obese according to the BCS score, additional analysis, including measurements of fat percentage and metabolic markers would improve the power of these correlations.

Changes in the ratio Firmicutes/Bacteroidetes seem to contribute to the development and preservation of obesity in dogs (Park et al., 2015). In agreement with humans and other animals, the increase in Firmicutes and decrease in Bacteroidetes generates an increase in the extraction of energy from the diet, mainly complex polysaccharides (Ley et al., 2006; Palmas et al., 2021). This has been suggested to lead to the induction of specific metabolic pathways involved in short-chain fatty acid production and finally causing an increase in adipose tissue in the individual (Martínez-Cuesta et al., 2021). Recently it has been shown by using metabolomics that weight loss in obese dogs induces several changes in fecal metabolites (Bermudez Sanchez et al., 2021). The actual contribution of alterations in the Firmicutes/Bacteroidetes and increased energy extraction to obesity has been challenged by several studies (Duncan et al., 2008; Schwiertz et al., 2010; Xiao & Kang, 2020). In addition, no studies have demonstrated that these alterations indeed contribute to obesity in dogs.

Of 119 genera found in the microbiota of these animals in this study, the *Bacteroides* genus was the most abundant in normal weight dogs. Comparatively, it showed a decrease in obese dogs (Fig. 3). These microorganisms carry important immunological and metabolic functions. They are related to healthy microbiomes in dogs and humans, participate in the production of IL-6 and IL-10, stimulating the expression of MHC class II (Tsuda et al., 2007). They are also major bacteria promoting the production of IgA in the large intestine (Schofield & Palm, 2018; Yang et al., 2020). *Bacteroides* species have been associated with the prevention of insulin resistance and correct energy metabolism (Rios-Covian et al., 2017; Gurung et al., 2020). They are believed to have a great therapeutic value in metabolic diseases such as diabetes and obesity (Yang et al., 2016). The role of Bacteroidetes in the gut microbiota of dogs has not been well studied, especially if they play similar roles as in the human gut.

The most abundant genera in obese dogs were *Peptoclostridium* and *Blautia* (Fig. 3). They belong to the *Clostridium* class and phylum Firmicutes. The increase in species of these genera has been related to certain disease states in dogs and humans, including obesity, metabolic syndrome, acute diarrhea, and IBD (Leung et al., 2013; Woting et al., 2014; Guard et al., 2015). For example, the *Blautia* genus has been related to visceral fat accumulation in adult humans between 20 and 76 years of age, independent of external factors such as diet (Ozato et al., 2019). Changes in certain KEGG categories here were associated with increases in *Blautia* and *Allobaculum*. Certain studies have shown this last genus to increase in high-fat diets in mice and dogs (Kilburn et al., 2020; Zheng et al., 2021).

Metabolic analyses have supported the hypothesis that microbial gut ecology creates functional changes that help perpetuate obesity (Backhed & Crawford, 2010). The microbiome of obese mice is enriched in genes that decode for the catabolism of complex polysaccharides, promoting higher absorption of polysaccharides from the diet and subsequent metabolism of monosaccharides (Turnbaugh et al., 2008). This precedes de novo lipogenesis (DNL), a hepatic pathway responsible for converting excess carbohydrates into fatty acids that are subsequently esterified to store triacylglycerols (TGs), providing energy for the energy pathway of β-oxidation of fatty acids (Ameer et al., 2014). It is believed that the increased absorption of polysaccharides from the diet occurs due to an increase in microbial glycosyl hydrolases present in multiple intestinal bacteria, including those belonging to *Bacteroidetes* and *Firmicutes*, increasing the transactivation of lipogenic enzymes and increasing the deposit of fat in peripheral tissues (Backhed et al., 2004).

In this study, we predicted the enrichment of KEGG orthologs K03406 and K07483 in the obese group. Previously, other authors have identified these genes in dogs with diarrhea compared with a healthy group (Guard et al., 2015). These genes, which code for methyl-accepting chemotaxis protein and transposases, are related to the formation of biofilms, biosynthesis of flagella, production of exopolysaccharides and toxins, among others. These changes are likely a reflection of the enrichment in pro-inflammatory, flagellated bacteria in the gut of obese animals, contributing to their obese phenotype (Salah Ud-Din & Roujeinikova, 2017).

## Conclusions

Obesity is a multifactorial disease highly prevalent in dogs. In this study, we compared the gut microbiome of normal weight and obese dogs. Their microbiome compositions were observed to be different. At the phylum level, obese animals showed an increase in Firmicutes (*Blautia*, *Peptoclostridium*) and a decrese in Bacteroidetes (*Bacteroides* spp). An increase in pathways related to motility and chemotaxis was observed in obese animals, which could contribute to their phenotype. It is essential to understand the contribution of specific microbiome taxa and their metabolic activities to obesity in dogs and how this information could be used in combination with diet to manage this disease.

## Supplemental Information

10.7717/peerj.12695/supp-1 Supplemental Information 1 Diet consumed by the animals in the study

10.7717/peerj.12695/supp-2 Supplemental Information 2 LEfSe analysis to determine genera enriched in obese and normal weight dogs

10.7717/peerj.12695/supp-3 Supplemental Information 3 Genus-level relative abundances Relative abundance at the genus-level in all animals in this study.

## Additional Information and Declarations

### DNA Deposition

The following information was supplied regarding the deposition of DNA sequences: The data is available at ENA: PRJEB38793.

### Data Availability

The following information was supplied regarding data availability: The relative abundances at the genus or ASV level is available in the Supplementary File.
