# Supplementary material for: Differences in the composition and predicted functions of the intestinal microbiome of obese and normal weight adult dogs
Source: PeerJ. 2022 Feb 16;10:e12695. doi: 10.7717/peerj.12695 (PMC8857902; doi:10.7717/peerj.12695)
Supplement: Supplemental Information 1 [file peerj-10-12695-s001.docx]

**Supplementary Table 1**: Diet consumed by the animals in the study

| **Code** | **Protein (%)** | **Fat (%)** | **Fiber (%)** | **Humidity (%)** | **Main ingredients in diet** |
| --- | --- | --- | --- | --- | --- |
| 1-N | 23 | 11 | 3,5 | 12 | corn, poultry by-product meal, meat and bone meal, soybean meal |
| 2-N | 21 | 9 | 4 | 10 | meat and bone meal, wheat flour, soy flour, rice |
| 3-N | 21 | 9 | 4 | 10 | meat and bone meal, wheat flour, soy flour, rice |
| 4-N | 21 | 9 | 4 | 12 | corn, poultry by-product meal, meat and bone meal, soybean meal |
| 5-N | 21 | 9 | 4 | 12 | corn, meat and bone meal, pork hydrolyzate, wheat |
| 6-N | 24 | 8 | 3,5 | 9 | wheat, soybean meal, chicken by-product meal, corn |
| 7-N | 25 | 12 | 4 | 12 | frozen chicken and lamb meat, corn, chicken by-product flour, wheat |
| 8-N | 26 | 15 | 3 | 12 | Frozen chicken meat, corn, chicken by-product meal, wheat |
| 9-N | 24 | 8 | 3,5 | 9 | wheat, soybean meal, chicken by-product meal, corn |
| 10-N | 22 | 18 | 4,7 | 10% | Beer rice, brown rice, chicken meal, chicken fat |
| 1-O | 21 | 9 | 4 | 12 | corn, meat and bone meal, pork hydrolyzate, wheat |
| 2-O | 21 | 9 | 4 | 12 | corn, meat and bone meal, pork hydrolyzate, wheat |
| 3-O | 26 | 15 | 3 | 12 | Frozen chicken meat, corn, chicken by-product meal, wheat |
| 4-O | 26 | 15 | 3 | 12 | Frozen chicken meat, corn, chicken by-product meal, wheat |
| 5-O | 23 | 11 | 3,5 | 12 | corn, poultry by-product meal, meat and bone meal, soybean meal |
| 6-O | 24 | 8 | 4 | 12 | wheat, soybean meal, chicken by-product meal, corn |
| 7-O | 21 | 9 | 4 | 10 | meat and bone meal, wheat flour, soy flour, rice |
| 7-O | 23 | 11 | 3,5 | 12 | corn, poultry by-product meal, meat and bone meal, soybean meal |
| 8-O | 25 | 12 | 4 | 12 | frozen chicken and lamb meat, corn, chicken by-product flour, wheat |
| 9-O | 25 | 12 | 4 | 12 | frozen chicken and lamb meat, corn, chicken by-product flour, wheat |
| 10-O | 24 | 8 | 4 | 12 | wheat, soybean meal, chicken by-product meal, corn |
