# Supplementary figures and images for: Differences in the composition and predicted functions of the intestinal microbiome of obese and normal weight adult dogs
Source: PeerJ. 2022 Feb 16;10:e12695. doi: 10.7717/peerj.12695 (PMC8857902; doi:10.7717/peerj.12695)

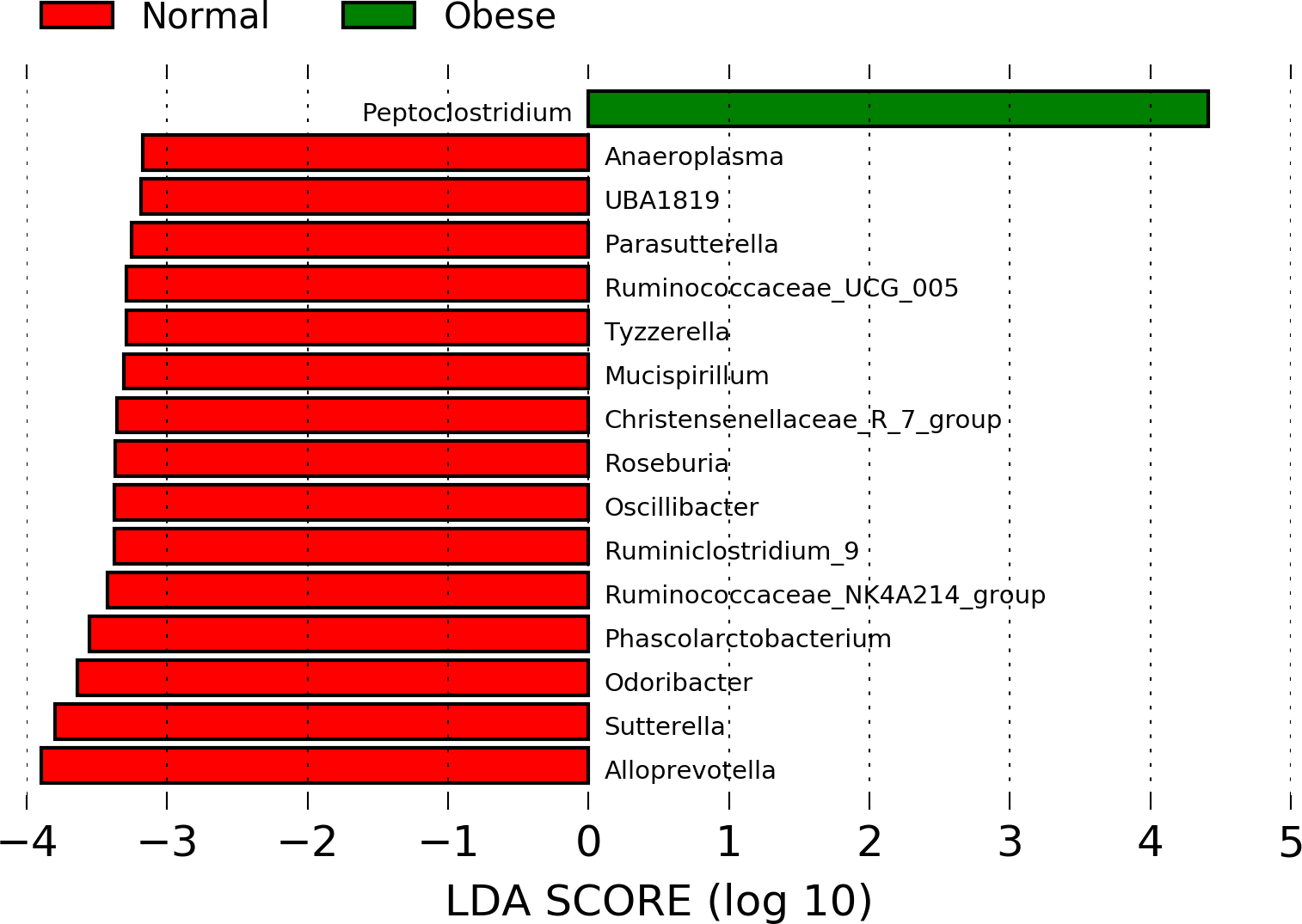

Supplement: Supplemental Information 2 [file peerj-10-12695-s002.png]
